# The Head-off Environmental Asthma in Louisiana (HEAL) Study—Methods and Study Population

**DOI:** 10.1289/ehp.1104239

**Published:** 2012-08-15

**Authors:** Patricia C. Chulada, Suzanne Kennedy, Mosanda M. Mvula, Katy Jaffee, Jeremy Wildfire, Eleanor Thornton, Richard D. Cohn, L. Faye Grimsley, Herman Mitchell, Jane El-Dahr, Yvonne Sterling, William J. Martin, LuAnn White, Kevin U. Stephens, Maureen Lichtveld

**Affiliations:** 1 Clinical Research Program, National Institute of Environmental Health Sciences, National Institutes of Health, Department of Health and Human Services, Research Triangle Park, North Carolina, USA; 2 Rho Federal Systems Division, Inc., Chapel Hill, North Carolina, USA; 3 New Orleans Health Department, New Orleans, Louisiana, USA; 4 Visionary Consulting Partners, LLC, Fairfax Station, Virginia, USA; 5 SRA International, Inc., Durham, NC, USA; 6 School of Public Health and Tropical Medicine, Tulane University, New Orleans, Louisiana, USA; 7 Department of Pediatrics, Tulane University School of Medicine, New Orleans, Louisiana, USA; 8 Health Sciences Center School of Nursing, Louisiana State University, New Orleans, Louisiana, USA; 9 National Institute of Child Health and Development, National Institutes of Health, Department of Health and Human Services, Bethesda, Maryland, USA

**Keywords:** asthma, asthma case management, asthma counselor, environmental intervention, Hurricane Katrina, indoor allergens, mold

## Abstract

Background: In the city of New Orleans, Louisiana, and surrounding parishes (NOLA), children with asthma were perilously impacted by Hurricane Katrina as a result of disrupted health care, high home mold and allergen levels, and high stress.

Objectives: The Head-off Environmental Asthma in Louisiana (HEAL) study was conducted to examine relationships between the post-Katrina environment and childhood asthma in NOLA and assess a novel asthma counselor intervention that provided case management and guidance for reducing home mold and allergen levels.

Methods: Children (4–12 years old) with moderate-to-severe asthma were recruited from NOLA schools. Over 1 year, they received two clinical evaluations, three home environmental evaluations, and the asthma intervention. Quarterly end points included symptom days, medication use, and unscheduled emergency department or clinic visits. A community advisory group was assembled and informed HEAL at all phases.

Results: Of the children (*n* = 182) enrolled in HEAL, 67% were African American, and 25% came from households with annual incomes < $15,000. HEAL children were symptomatic, averaging 6.6 symptom days in the 2 weeks before baseline, and had frequent unscheduled visits to clinics or emergency departments (76% had at least one unscheduled visit in the preceding 3 months). In this report, we describe study design and baseline characteristics of HEAL children.

Conclusions: Despite numerous challenges faced by investigators, study staff, and participants, including destroyed infrastructure, disrupted lives, and lost jobs, HEAL was successful in terms of recruitment and retention, the high quality of data collected that will provide insight into asthma-allergen relationships, and the asthma intervention. This success was attributable to using an adaptive approach and refining processes as needed.

Hurricane Katrina struck the city of New Orleans and surrounding parishes (NOLA) on 29 August 2005 and was one of the most destructive and costly disasters in U.S. history. Storm surges breached substandard flood walls along outfall canals, and as a result, 80% of Orleans Parish and large tracts of land in neighboring parishes flooded (Kates et al. 2006). Water rose to 20 feet in some locations and remained for weeks, damaging approximately 70% of housing, displacing approximately 1 million residents, and destroying much of the city’s infrastructure (Liu et al. 2006). The environmental consequences of the flooding and rain included a copious growth of mold and other microbes and increased levels of asthma triggers. The Centers for Disease Control and Prevention and the Louisiana Department of Health and Hospitals (DOHH) found that 46% of 112 homes examined in the aftermath of Katrina had visible mold, with 17% having heavy mold contamination (Rao et al. 2007; Ratard et al. 2006). The levels of mold by-products (β-d-glucans) found in these homes have been associated with cough, nose/throat irritation, decreased lung function, tiredness, and headache (Rylander and Lin 2000; Rylander et al. 1998). Other homes in the same study had levels of bacterial by-products (endotoxin) that have been associated with decreased pulmonary function (Douwes et al. 2002; Reynolds et al. 2001).

In addition to the environmental devastation, Katrina destroyed an already strained health care system and its provider base. About 4,500 physicians left the region, leaving 50% of children who previously had a health care provider without one. Most of Orleans Parish’s hospitals and clinics were closed, including Charity Hospital, where 90% of the poor and uninsured received medical care (Rudowitz et al. 2006). Numerous pharmacies were destroyed, and residents had difficulty filling prescriptions because of lost or missing medical records, insurance cards, and Medicaid cards (Henry J. Kaiser Family Foundation 2007).

In 2006, the Merck Childhood Asthma Network and the National Institute of Environmental Health Sciences (NIEHS) formed a public–private partnership that later joined with the National Institute of Minority Health and Health Disparities and local institutions [New Orleans Health Department (NOHD) and Tulane and Louisiana State Universities] to conduct the Head-off Environmental Asthma in Louisiana (HEAL) Study. They viewed post-Katrina NOLA as an unprecedented opportunity to study relationships between environmental triggers and childhood asthma morbidity while simultaneously addressing the high rate of childhood asthma morbidity in this region. Asthma, like most chronic diseases, is influenced by the quality and continuity of health care. Ongoing monitoring of symptoms and medication use are crucial in its management. The disruption to health care by Katrina put children with asthma at risk, which was exacerbated by prolonged exposures to environmental hazards that affect symptoms (Rao et al. 2007; Ratard et al. 2006; Solomon et al. 2006).

HEAL was an observational study designed to examine relationships between childhood asthma morbidity and the physical (allergens, mold, other exposures) and psychosocial (stress, social support, health care barriers) impacts of the post-Katrina environment. Another goal was to implement and assess a novel asthma counselor intervention that provided both asthma case management and guidance for environmental remediation. The intervention drew upon the National Cooperative Inner-City Asthma Study (NCICAS) (Evans et al. 1999) and the Inner-City Asthma Study (ICAS) (Crain et al. 2002; Morgan et al. 2004) but contained some unique aspects. NCICAS was initiated in 1994, conducted in eight major U.S. inner cities, and encompassed 1,033 children with asthma. The children were randomized into two groups: a control group that received “usual asthma care,” and an intervention group that received asthma case management for 1 year in addition to “usual asthma care.” In the intervention group, the children’s caretakers also received education on asthma triggers, environmental controls, asthma physiology, and techniques for better communicating with their children’s physicians. ICAS was initiated in 1998, conducted in seven major inner cities, and encompassed 937 children with asthma. Similar to NCICAS, the children were randomized into two groups. Both groups received “usual asthma care,” but the children in the intervention group also received home remediation focused on reducing/eliminating exposure to cockroach, dust mite, rodent, pet, and mold allergens in addition to environmental tobacco smoke. In conducting HEAL, we combined aspects from both NCICAS and ICAS into a hybrid intervention with modifications to meet the postdisaster needs of NOLA families. We believed this approach would result in a field-applicable model for other disaster situations. In this report, we describe the methods, implementation, and study population of HEAL.

## Methods

**Figure 1:**
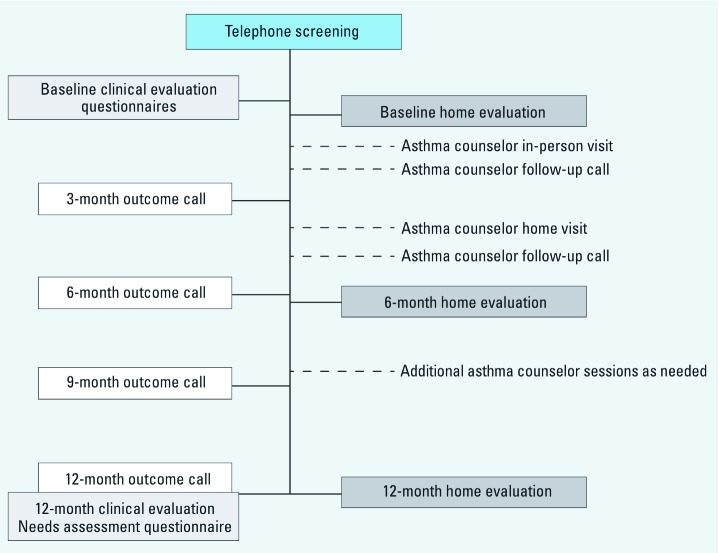
Timeline for HEAL study activities. Children were prescreened for eligibility by phone. During the call, caretakers were asked about the children’s asthma symptoms and post-Katrina living conditions. Eligible children were invited to have a baseline clinical evaluation, where they received a complete asthma workup to confirm their asthma status, blood work for basic clinical and immunological parameters, and skin sensitivity testing. Children who were enrolled in HEAL received three home evaluations and the hybrid asthma counseling intervention over a 1-year period. During each home evaluation, the children’s homes were examined visually, and air and dust samples were collected. The intervention included two in-person visits with an asthma counselor, with one of these conducted in the children’s homes, and additional asthma counseling sessions as needed. The asthma counselor called the caretaker 2 weeks after each in-person session to provide follow-up. To assess changes in the children’s asthma morbidity, caretakers were called every 3 months and surveyed about their children’s asthma outcomes. At the end of the 1-year study period, the children received a final clinical evaluation.

*Study design.* HEAL was a pre–post interventional study that aimed to *a*) characterize relationships between environmental exposures and childhood asthma morbidity in post-Katrina NOLA; *b*) assess a novel asthma counselor intervention; and *c*) collect biological and environmental specimens to support future studies. Children with moderate-to-severe asthma were recruited from NOLA schools, prescreened by telephone, and clinically evaluated to confirm eligibility and collect baseline data. Over 1 year, they received three home environment evaluations, asthma counseling, and another clinical evaluation. Asthma morbidity end points were collected quarterly (Figure 1). HEAL was approved by the NIEHS, Tulane University, and Louisiana State University institutional review boards.

*Population.* Children were 4–12 years old, lived in NOLA, and had moderate-to-severe asthma as defined by the 2007 National Asthma Education and Prevention Program (National Heart, Lung, and Blood Institute 2007). Caretakers were the children’s legal guardians. Children were required to sleep in the intervention home ≥ 5 nights/week. Children were excluded if they had a hypoxic seizure or a life-threatening asthma exacerbation in the preceding 5 years requiring intubation or mechanical ventilation, if they had a serious medical condition other than asthma, or if their caretaker planned to move from NOLA during the year.

*Recruitment*. During the first 6 months of recruitment (February–August 2007), children in pre-kindergarten to grade 6 in public and parochial schools in Orleans Parish were targeted. They were given a one-page letter to bring home to their caretakers. The letter, on school letterhead and signed by the school principals, asked caretakers about their children’s symptoms and whether they were interested in participating in a study about asthma. Children were instructed to return the letters to their teachers, indicating whether caretakers were interested. Some schools conducted recruitment internally, using their own staff (trained by HEAL staff). Other schools were inadequately staffed and let HEAL staff distribute the letters. Similar letters were also given to children attending school-sponsored summer camps in 2007. During the second 6 months of HEAL (September 2007–March 2008), recruitment was expanded to schools in four other parishes (Jefferson, St. Tammany, St. Bernard, and Plaquemines Parishes). In addition, the procedure for distributing letters was modified. Instead of distributing letters to all children in the classroom to take home to caretakers, recruitment letters were now written by school nurses and distributed only to children in the classroom with documented asthma to take home to caretakers. Nonresponders received follow-up letters and telephone calls, also from school nurses.

Information about HEAL was also disseminated through local radio and television stations, newspapers, and magazines. Pamphlets and posters were placed in schools, churches, community centers, pharmacies, clinics, emergency departments (EDs), and physician’s offices. Community advisory group (CAG) members (described below) attended local events to discuss HEAL and pass out pamphlets.

*Prescreening*. Caretakers interested in HEAL were prescreened with a 15-min phone survey by trained nurses from Tulane’s Clinical and Translational Research Center. They were asked about their children’s asthma symptoms, medication usage, ED visits, hospitalizations, and current living conditions. Eligible children were invited to the baseline clinical evaluation.

*Baseline.* At the baseline clinical evaluation, trained research assistants described HEAL to participants and answered their questions. Children were examined by physicians board-certified in pediatrics and in allergy and immunology for atopy and to confirm moderate-to-severe asthma. If final eligibility was met, caretakers were asked for written informed consent. Children were asked for oral (if < 7 years of age) or written (if ≥ 7 years of age) assent. Other baseline procedures included taking medical histories (emphasizing respiratory symptoms and medications), pulmonary function testing (PFT) (spirometry on children 6–12 years of age and peak flow on all children), and blood collection (16 mL) by venipuncture. Blood was used for complete blood cell counts and differentials, serum levels of total and allergen- and mold-specific IgE, and archiving for future studies.

Allergen skin testing was performed using a multi-test device (Multi-Test II; Lincoln Diagnostics, Inc., Decatur, IL) applied to the volar surface of the arms. The panel included dust mites (Der p and Der f mix), cockroach (American and German mix), cat, dog, mouse, rat, Bermuda grass, and molds (*Alternaria, Cladosporium, Aspergillus fumigatus, Penicillium,* and 10 other molds found in high concentrations in NOLA).

Caretakers completed questionnaires for literacy, quality of life, children’s behavior, attitudes about and knowledge of asthma, health care access and barriers, life events, and stress. All questionnaires in HEAL were administered to caretakers by trained research assistants, except for one that was validated as a self-administered questionnaire. Caretakers were also interviewed about their smoking habits and their children’s symptoms, unscheduled ED or clinic visits, hospitalizations, medications, adherence, and home environments. Children completed verbal or written questionnaires, depending on age, about asthma attitudes and quality of life.

The baseline clinical evaluation took approximately 3 hr to complete. Afterward, the children’s primary care physicians were sent letters summarizing the results.

*Final clinical evaluation.* Another clinical evaluation was conducted approximately 12 months after the baseline examination; this consisted of a physical examination, PFT, and blood collection (10 mL) for measuring serum levels of total and mold-specific IgE.

The questionnaires used at baseline were re-administered to caretakers by the trained research assistants during the 12-month follow-up. In addition, caretakers were administered a new post-Katrina needs assessment questionnaire that contained questions from the Kaiser Report (Henry J. Kaiser Family Foundation 2007) and the Breslau posttraumatic stress disorder scale (Breslau et al. 1999) as well as new questions developed by HEAL investigators based on their post-Katrina experiences. These questionnaires focused on stress and the emotional, psychological, and physical impacts of Katrina, ongoing problems, support systems, and needs.

*Home environmental evaluations.* Homes of HEAL children were evaluated at baseline and at 6 and 12 months by trained technician pairs. If a child relocated within 6 months after the baseline evaluation, the baseline evaluation was repeated in the new home. Evaluations focused on environmental hazards and allergens, and consisted of visual inspections, dust and air sampling, and face-to-face surveys of caretakers. The survey was a modified version of the ICAS survey that was supplemented with questions from the U.S. Department of Housing and Urban Development/NIEHS National Survey of Lead Hazards and Allergens in Housing (NIEHS 2002).

During the indoor inspection, technicians recorded evidence of moisture, water damage, environmental tobacco smoke, pests (cockroaches, rodents), mold, and other hazards. Air samples were collected from the living room, children’s sleeping area/bedroom, and outside the home using Air-O-Cell spore traps (Zefon International, Ocala, FL). These samples were analyzed for mold (total fungal spores and > 30 taxa categorizations). Dust samples were collected from the family/living/television room, kitchen, and children’s bedroom floor and bed (Gruchalla et al. 2005). Bed dust was analyzed for Der p 1 (dust mite), Bla g 1 (cockroach), Mus m 1 (mouse), and *Alternaria* allergens. If bed dust samples were insufficient for testing, they were supplemented or replaced with bedroom floor dust. Remaining bed and floor dust was analyzed for endotoxin and (1□3) and (1□6)-β-d-glucans (laboratory of Peter Thorne, Environmental Health Sciences Research Center, University of Iowa) (Blanc et al. 2005). Dust from the family/living/television room was combined with bedroom dust and analyzed by polymerase chain reaction (PCR) for mold species (Vesper et al. 2004, 2007).

During one of the home environment evaluations, technicians administered a one-time questionnaire (Home Remediation and Remodeling Questionnaire) to caretakers, which asked about post-Katrina renovations and mold remediation. Additional details about these evaluations are provided in the accompanying article (Grimsley et al. 2012).

*Asthma counselor intervention*. The goals of the asthma counselor intervention were to empower caretakers to better manage their children’s symptoms, improve interactions with primary care providers, reduce home allergen levels, and manage psychosocial issues and stress resulting from Katrina. The intervention combined case management elements from NCICAS (Evans et al. 1999) and environmental remediation measures from ICAS (Crain et al. 2002; Morgan et al. 2004) into a hybrid model that also incorporated modifications to address postdisaster problems. These included helping caretakers identify health care centers, identify pharmacies and providers, navigate public services established for post-Katrina victims, and replace lost documents such as insurance and prescription cards. Evidence-based practices incorporated into HEAL included the Child Asthma Risk Assessment Tool (CARAT) (Evans et al. 1999) and Environmental Risk Assessment Tool (ERAT) (Crain et al. 2002). These are computer-generated reports that estimate children’s risk levels based on the results of their clinical examinations, allergen sensitivities, and environmental exposures.

Most asthma counselors had master’s degrees in health-related fields, backgrounds in counseling and public health, and experience working in community programs. They worked in teams with community health workers, who were high school graduates, worked for community-based organizations, and were familiar with NOLA communities. The teams underwent rigorous training and had lighter caseloads compared with NCICAS counterparts. Additional details about the asthma counselor model are provided in the accompanying article (Mitchell et al. 2012).

*Compensation and retention.* Participating caretakers received gift cards for completing the baseline clinical evaluation ($50), 12-month clinical evaluation ($40), home evaluations ($15 for each), and quarterly phone assessments ($15 for each). Caretakers received an additional $40 at the completion of the study.

Measures were taken to facilitate participation and minimize attrition. Caretakers were asked to update their contact information and that for family members and friends who served as back-ups at every point of HEAL contact (by asthma counselors, by home evaluators, by administrators of the quarterly surveys, and every time a HEAL caretaker contacted HEAL staff). Evening and weekend appointments were offered to accommodate caretakers’ schedules. Transportation to and from appointments was prearranged and subsidized.

*Community engagement.* A CAG was assembled to engage local constituents and consisted of 15 members, including parents, individuals representing schools, faith-based and community groups, local businesses, medical clinics, and media. CAG members facilitated communication between communities and investigators, incorporated cultural and community views into HEAL, helped develop recruitment strategies and resolve problems, and represented HEAL at community events. The CAG met periodically with investigators, received reports on study progress, and disseminated results to local communities.

*Health outcomes.* Health outcomes data were collected from caretakers at baseline and 3, 6, 9, and 12 months after baseline using a brief (< 5 min) phone survey. The primary outcome was maximum symptom days (MSDs) for asthma. MSDs are determined as the number of days of the asthma symptom with the highest number of occurrences in the preceding 2 weeks. The different asthma symptoms were defined as *a*) the number of days with wheezing, chest tightness, and cough; *b*) the number of nights with disturbed sleep resulting from asthma; or *c*) the number of days of disrupted activities because of asthma. Secondary outcomes included the number of unscheduled ED and clinic visits due to asthma in the previous 3 months, the number of hospitalizations due to asthma in the previous 3 months, and the number of prednisone bursts (short high-dose steroid treatments used to calm asthma symptoms) in the previous month. The same outcomes also were assessed during clinical evaluations.

*Statistical methods.* We compared the characteristics of children from Orleans and Jefferson Parishes using *t*-tests (continuous outcomes) and Fisher’s exact test (categorical outcomes). Parish-specific seasonal trends for symptoms and mold levels were analyzed using locally estimated scatterplot smoothing (LOESS) curves with 95% confidence intervals. Analyses were conducted using SAS version 9.2 (SAS Institute Inc., Cary, NC) and R version 2.11.1 (R Project for Statistical Computing, Vienna, Austria).

## Results

*Study design and recruitment.* HEAL was initially designed as a randomized, controlled trial requiring 450 children to detect a significant reduction in asthma symptoms. Specifically, children with moderate-to-severe asthma were to be recruited from Orleans Parish over 6 months (March–August 2007) and randomized either to a control group (*n* = 225) that would receive an excellent standard of care or to an intervention group (*n* = 225) that would receive the same standard of care plus the asthma counselor intervention. However, because only 77 children had been enrolled after 6 months, starting in September 2007 we expanded recruitment into four other parishes (Jefferson, St. Bernard, St. Tammany, and Plaquemines Parishes) and extended recruitment for an additional 6 months (September 2007 through February 2008). In addition, instead of distributing recruitment letters to all children in the classroom to bring home to caretakers, recruitment letters were now written by school nurses and distributed only to children with documented asthma to take home to caretakers.

Over the entire 1-year recruitment period, 182 children were recruited into HEAL. A total of 105 children were recruited into HEAL during the second 6 months after the recruitment modifications were implemented. Of these 105 children, 49 came from Orleans Parish, 49 came from Jefferson Parish, 5 came from St. Bernard Parish, and 2 came from St. Tammany Parish. Children enrolled from all parishes were included in the study and overall analyses. In the comparison between parishes (Table 1), we included the 7 children from St. Bernard and St. Tammany Parishes in with the children from Jefferson Parish.

**Table 1:** Baseline demographics and housing characteristics of HEAL children by parish (%).

All (*n* = 182)	Orleans Parish [*n* = 126 (69%)]	Jefferson Parisha [*n* = 56 (31%)]	*p*-Value
Demographics
Percent male	54	56	48	0.34
Race/ethnicity	< 0.01
African American	67	81	36
Hispanic	7	6	9
Caucasian/other	26	13	55
At least one household member employed	91	90	95	0.40
Household income < $15,000	25	31	12	< 0.01
At least one smoker in household	32	34	27	0.39
Total no. of people in household	0.73
2–3	29	31	25
4–5	57	56	59
≥ 6	14	13	16
Caretaker married	54	45	75	< 0.01
Caretaker completed high school	88	88	89	0.99
Housing
No. of times relocated since Katrinab	3.09 ± 2.03	3.26 ± 2.09	2.71 ± 1.85	0.09
Current housing type
Single-family detached house	64	56	82
Multifamily house (duplex/triplex/row house)	23	30	7
Apartment	8	8	9
FEMA trailer	5	6	2
Current housing damage	0.09
Flooding only	23	29	13
Roof leak only	25	23	29
Flooding and roof leak	14	14	12
None	38	34	46
Mold air samplingc
Indoor total	502	567	379	0.05
Outdoor total	3,958	4,054	3,751	0.74
aIncludes children recruited from Jefferson (49), St. Bernard (5), St. Tammany (2), and Plaquemines (0) parishes. bMean ± SD. cSpores/m3 reported as geometric means.								

Despite the study modifications implemented above, we still realized after the first 6 months that we would not be able to recruit enough children to support the original randomized, controlled trial study design. Therefore, HEAL was transitioned to the observational study described in “Methods.” This new design reduced the number of observations required to analyze study questions with adequate power (< 160 children for most comparisons). At the same time, the original study goals were maintained, the data already collected were used, and the impact on enrolled participants was minimized.

**Figure 2:**
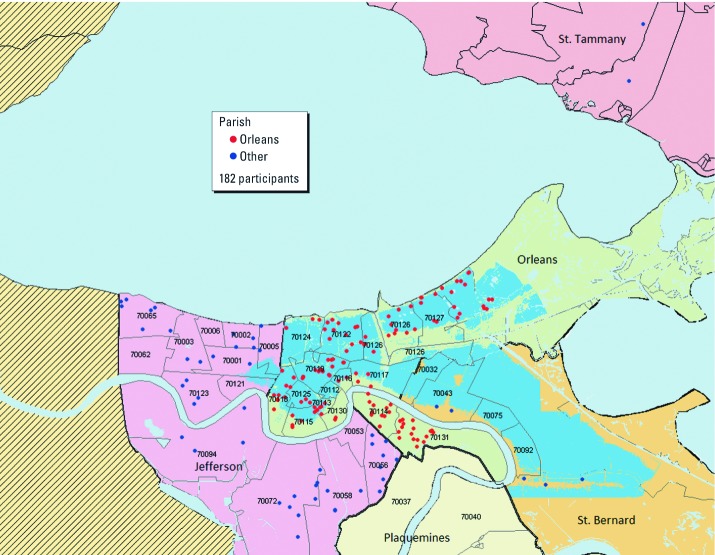
Geospatial mapping of the children’s homes at the time of study enrollment (*n* = 182 participants). Red dots ­represent children’s homes in Orleans Parish, and blue dots represent homes in Jefferson and other parishes (St. Bernard and St. Tammany). Flooding overlays were only available for Orleans and St. Bernard Parishes. Although areas of Jefferson Parish also flooded, they are not shown on this map. Numbers ­represent the children’s ZIP codes.

Over the 1-year recruitment period, 184 schools participated, including 89 schools in Orleans Parish and 95 in Jefferson and other parishes. More than 36,000 school letters were distributed, and 6,911 were returned. From this response and responses from other recruitment methods (e.g., disseminating study material through local news media outlets, distributing pamphlets and posters, and community engagement), a total of 2,821 children were found eligible for a phone screen. Of these, 1,864 were successfully prescreened, and 320 children who met prescreening criteria were invited to the baseline clinical evaluation. Baseline clinical evaluations were completed by 201 children; 193 of these met final eligibility requirements, and 182 were enrolled. Most of the enrolled children (89%) were recruited through schools, but some (11%) came from the other recruitment methods described above. The locations of the participating children’s homes, along with the areas affected by post-Katrina flooding, are shown in Figure 2.

*Demographics.* Children in HEAL were predominantly African American (67%), and about 25% came from households with annual incomes < $15,000 (Table 1). Demographic characteristics differed by parish. Orleans Parish children were predominantly African American (81%); Jefferson Parish children were predominantly Caucasian (55%). More Orleans Parish children came from households with annual incomes < $15,000 (31%) compared with Jefferson Parish (12%). However, in each parish, African Americans were more likely to come from households with annual incomes < $15,000 compared with Caucasians and other races (including those of Hispanic descent) (*p* < 0.001), and within-race income levels were similar for the two parishes (*p* = 0.95) (data not shown).

*Housing*. Families relocated an average of three times since Katrina (Table 1). Most (64%) were living in single-family dwellings; 5% were living in Federal Emergency Management Agency (FEMA) trailers. Most homes (62%) were damaged from Katrina, with 23% from flooding, 25% from roof leaks, and 14% from both flooding and roof leaks. Mean indoor airborne mold levels (502 spores/m^3^) were lower than outdoor levels (3,958 spores/m^3^). Mean indoor airborne mold levels were significantly higher in Orleans Parish homes than in Jefferson Parish homes (567 vs. 379 spores/m^3^) (*p* = 0.05).

*Health care access.* Most children (85%) had a usual place of asthma care, but parish differences were observed. Only 80% of Orleans Parish children had a usual place of care, compared with 96% of Jefferson Parish children (*p* < 0.01). Orleans Parish children also used EDs four times more often than Jefferson Parish children (15% vs. 3%, *p* = 0.05) (Table 2).

**Table 2:** Baseline access to care and morbidity of HEAL children (%) by parish.

All (*n* = 182)	Orleans Parish [*n* = 126 (69%)]	Jefferson Parisha [*n* = 56 (31%)]	*p*-Value
Access to care
Usual place for follow-up asthma care for child	85	80	96	< 0.01
Pre-Katrina: location of asthma care	< 0.01
Emergency department	13	17	2
Clinic/office	74	70	84
Both	13	13	13
Previous 12 months: location of asthma care	0.06
Emergency department	12	15	3
Clinic/office	76	72	86
Both	12	13	11
Financial/insurance problems affecting asthma meds	12	14	9	0.46
Baseline morbidity
Symptoms 2 weeks before baseline
MSDsb	6.64 ± 4.86	6.17 ± 4.71	7.70 ± 5.06	0.05
Days of wheeze	5.34 ± 4.54	4.81 ± 4.27	6.52 ± 4.92	0.02
Days child slowed down/stopped play	3.22 ± 3.93	3.10 ± 3.78	3.48 ± 4.27	0.55
Nights child woke due to asthma	3.29 ± 4.16	3.31 ± 4.14	3.23 ± 4.25	0.91
School days missed due to asthma	24	23	27	0.58
Caretakerb
Nights caretaker woke due to child’s asthma	2.99 ± 4.35	3.15 ± 4.46	2.63 ± 4.11	0.45
Days caretaker changed plans	0.88 ± 1.66	0.85 ± 1.59	0.95 ± 1.82	0.72
Lung functionb
Percent predicted FEV1	91.05 ± 16.74	92.83 ± 16.25	88.34 ± 17.34	0.21
FEV1/FVC ratio	78.22 ± 10.13	79.50 ± 8.79	76.28 ± 11.75	0.13
Health care utilizationc
At least 1 unscheduled visit (ED or clinic)	76	72	86	0.06
At least 1 prednisone burst	19	17	23	–0.49
At least 1 hospitalization due to asthma	3	2	5	0.37
aIncludes children recruited from Jefferson (49), St. Bernard (5), St. Tammany (2), and Plaquemines (0) parishes. bMean ± SD. cHospitalizations and unscheduled visits in the previous 3 months. Prednisone in the previous 1 month.								

*Baseline asthma morbidity and allergic sensitization.* HEAL children were symptomatic, averaging 6.64 MSDs; wheeze was the most common symptom, averaging 5.34 days in the previous two weeks (Table 2). Average FEV_1_ (forced expiratory volume in 1 sec) levels were generally normal (91.05 ± 16.74% predicted), but the average FEV_1_/FVC (forced vital capacity) ratio (78.22 ± 10.13) in HEAL children provided evidence for physiologic airway obstruction. Of the 182 children, 19% had an FEV_1_/FVC ratio < 70%, indicating compromised pulmonary function. For allergic sensitization, 89% of children skin-tested positive to at least 1 of 22 allergens; 72% tested positive to at least 1 of 14 molds.

Baseline morbidity differed between parishes. Overall, Jefferson Parish children had more MSDs (7.70 ± 5.06 vs. 6.17 ± 4.71; *p* = 0.05), days of wheeze (6.52 ± 4.92 vs. 4.81 ± 4.27; *p* = 0.02), and unscheduled ED or clinic visits (*p* = 0.06) than Orleans Parish children. However, when we compared these same baseline morbidity variables between Orleans and Jefferson Parishes during the second 6 months of recruitment only (August 2007 to March 2008), when recruitment was occurring concurrently in both parishes, there were no significant parish differences in MSDs, wheeze, and unscheduled ED or clinic visits.

*Study progress and attrition.* Study activities concluded in September 2009. Of the 182 children enrolled, 161 (88%) completed the study; of those who did not, 5 withdrew, 3 moved out of NOLA, and 13 were lost to follow-up.

## Discussion

Hurricane Katrina caused catastrophic environmental devastation and disrupted the lives of people in NOLA. Flooding and rain destroyed much of the area’s infrastructure, and residents suffered great losses, including homes, jobs, schools, support systems, and sometimes their lives. Concerns included the lack of health care and rising mold and allergen levels and their potential effects on children with asthma. HEAL was designed to characterize post-Katrina exposures of children with asthma and address their health care management. However, numerous challenges from the devastation were faced when implementing HEAL. Approximately 1 million residents had evacuated the area, and only about 50% had returned by mid-2007 (Elliot 2009). Vague population estimates made it difficult to develop a study framework and estimate recruitment and statistical power for various study designs. Furthermore, we encountered problems in recruiting children, which resulted in low numbers. This difficulty might have been caused by a low number of children with asthma in general (many children with asthma might not have returned to NOLA after Katrina) or by other reasons. Given the vague population estimates, the reason for low recruitment is unclear. Despite these challenges, however, HEAL was a success in terms of the high quality of data collected, which will provide insight into postdisaster asthma–allergen relationships. These include data characterizing postdisaster exposures and asthma morbidity, but more importantly, data on effective interventions aimed at reducing postdisaster asthma symptoms. HEAL’s success derived from taking an adaptive approach and refining processes as needed, as described below.

An important modification that epitomizes the adaptive approach used in HEAL, and ultimately led to its success, was transitioning HEAL from a clinical trial to a 1-year observational study with postintervention follow-up. HEAL was initially designed as a randomized, controlled trial, but after 6 months, it became obvious that the recruitment numbers would not support this design. This problem was presented to the Data and Safety Monitoring Board, who worked with the investigators to modify the design and maintain the original study goals. With the new study design, data collection (morbidity, skin sensitivity, exposures) remained the same, and the quarterly end points could still be used to examine relationships between morbidity and exposures and to assess the effectiveness of the asthma counselor intervention.

Another important modification that led to HEAL’s success was refined recruitment strategies. These refinements, especially the nurse-based recruitment, were effective and led to increased enrollment during the second 6 months. Nurse-based recruitment allowed us to target children with documented asthma, enabling more focused messaging that resonated effectively with caretakers. It also supported more efficient eligibility screening as interviewers worked with an asthma-enriched population rather than children with broad respiratory conditions. Other recruitment strategies (media, pamphlets, community engagement) raised public awareness of HEAL but resulted in only a small increase in enrollment (approximately 11%). Thus, active recruiting methods proved more successful in this environment, but passive recruiting helped to increase numbers somewhat. Ultimately, 182 children were enrolled in HEAL. Despite all the post-Katrina challenges faced by NOLA residents and HEAL scientists, this is one of the largest childhood asthma populations ever recruited from a single geographic location compared with other National Institutes of Health–funded childhood asthma studies.

Unfortunately, the enrollment expansion introduced parish-related demographic differences. Children from Jefferson Parish were predominantly Caucasian and from higher-income households compared with Orleans Parish children. Surprisingly, Jefferson Parish children were more symptomatic (higher number of MSDs and days of wheeze). This is contrary to historical data showing that minority and low-income children with asthma are more symptomatic (Boudreaux et al. 2003; Erickson et al. 2007). However, the expansion into Jefferson and other parishes was initiated during the cold and flu season when asthma symptoms are typically elevated (Gergen et al. 2002). We also switched to nurse-based recruitment about the same time. These two modifications might have resulted in recruiting a more symptomatic population during the second 6 months. Furthermore, the statistically significant parish differences in MSDs and days of wheeze were eliminated when only children recruited concurrently from both Orleans and Jefferson Parish (during August 2007–March 2008) were compared (*p* = 0.53 for MSDs and *p* = 0.24 for wheeze) (data not shown). This result suggests that the differences may have been attributable partly to seasonal variation in asthma symptoms rather than actual differences between the two parishes.

One challenge of HEAL was the many activities required of participants in a postdisaster situation. Participants were required to take part in multiple, time-consuming activities over a short period of time during an otherwise stressful time in their lives. City infrastructure was still in disarray. Key public services (schools, transportation, utilities) were operating at less than half capacity, housing was scarce and expensive, unemployment was high, and federal money had not been distributed to areas most affected by Katrina (Liu et al. 2006). At the time of HEAL, 32% of NOLA residents said their lives remained “very disrupted” or “somewhat disrupted”; this value rose to 59% among African Americans (Henry J. Kaiser Family Foundation 2007). In addition, hiring and maintaining study staff was problematic as potential hires experienced similar problems. We overcame these problems because of a highly dedicated staff and the strong desire of caretakers to alleviate their children’s symptoms, which can be attested to by the high percentage of participants who completed the study.

HEAL children were more symptomatic at baseline (6.64 MSDs) compared with children from NCICAS (MSDs = 5.18) and ICAS (MSDs = 5.99). HEAL children also had more unscheduled ED or clinic visits in the previous 3 months (76%) compared with NCICAS (30%) and ICAS (51%) children—which was surprising because HEAL children were better off economically. Only 25% of HEAL children came from households with annual incomes < $15,000, whereas 67% and 60% of NCICAS and ICAS children, respectively, came from households with annual incomes < $15,000. The lower economic situations of NCICAS and ICAS children were a result of recruitment strategy; NCICAS and ICAS targeted children living in census tracks where > 20% of households had an annual income below the federal poverty line. In HEAL, children were targeted based on residing in an area impacted by Katrina; having a specific annual income was not an eligibility requirement. Although the economic differences between the study populations resulted from differing recruitment strategies, the morbidity differences were real and require further examination. The higher morbidity in HEAL, despite the children’s better economic circumstances, might be attributable to living in a postdisaster region with increased environmental exposures, high stress, and adverse psychosocial factors. It is well recognized that such factors have a strong impact on childhood health (Turyk et al. 2008; Wright 2005).

Characterizing post-Katrina exposures and exploring their relationships to asthma was an important focus of HEAL that will be described in future publications. Testing for mold–asthma relationships is problematic because of limitations of mold methodologies and the lack of biomarkers (Brandt et al. 2006). Typically, mold is cultured from air (colony-forming units per cubic meter), but this procedure does not detect nonviable and unculturable species, resulting in exposure underestimations because nonviable mold also carries mycotoxins that can trigger allergic reactions. Dust has been proposed as a better medium for mold analyses and might represent longer timeframes of potential exposures; when analyzed by PCR, nonviable and unculturable species in dust can be detected. In HEAL, we used a comprehensive, multipronged approach to characterize mold and other exposures. Different samples (air, dust, HEPA filter extracts) were collected and examined for many molds, allergens, β-d-glucans, and endotoxin. When analyzed in conjunction with specific biomarkers (skin sensitivities, serum IgEs), these data will provide us with important insights into asthma–allergen–mold relationships. HEAL baseline findings of indoor mold and allergens are described in the accompanying article (Grimsley et al. 2012).

In addition to studying environmental relationships with asthma in a postdisaster setting, another goal of HEAL was to intervene in childhood asthma morbidity using a novel hybrid asthma counselor model that combined traditional case management with education and aid in reducing home exposures. We felt that incorporating allergen reduction measures in the model might increase its effectiveness considering the levels of mold, allergens, and other toxic exposures that were reported following Katrina. We found that HEAL children were highly sensitive to molds compared with children in earlier studies. In ICAS, 100% of children were skin-test positive (children were required to test positive to at least one of nine allergens to be enrolled; four of the nine were molds). In HEAL, 89% of children were skin-test positive (children were not required to be skin-test positive). However, only 50% of ICAS children tested positive for one of the four mold species (O’Connor et al. 2004), whereas 67% of the skin test–positive children in HEAL tested positive for at least one of the same four species.

## Conclusion

HEAL will provide valuable insight into postdisaster asthma–allergen relationships. HEAL was successful despite the extreme challenges faced by investigators in conducting HEAL and by participants in dealing with competing demands. These challenges were overcome by taking an adaptive approach that included refining recruitment processes, being flexible in scheduling, cross-communicating between study teams for the diligent follow-up of participants, and identifying and subsequently augmenting resources in problem areas. HEAL also underscores the importance of involving community members in the research process. CAG members represented diverse segments of the community, including schools, religious groups, health care providers, and parents. They helped to anticipate problems in implementing HEAL and provided insight into research design, recruitment and implementation, psychosocial and socioeconomic issues, and cultural norms.

The success of HEAL is measured in its sustainability. Pilot programs/studies are rarely sustained for multiple reasons, such as problems with buy-in and maintaining funding and the interest of supporters. However, because of its success and support from Merck Childhood Asthma Network, the HEAL hybrid asthma counselor model has been adopted by multiple public health programs, including those administered by Xavier University’s Center for Minority Health and Health Disparities Research and Education Program in NOLA, George Washington’s School of Public Health and Health Services, and Rho, Inc., at federally qualified health centers in the United States.

## Appendix

HEAL was a collaboration of the following institutions, investigators, and staff: Tulane University School of Public Health and Tropical Medicine, Maureen Lichtveld (principal investigator), Faye Grimsley (investigator), LuAnn White (investigator), William Hawkins (program management), Melissa Owsiany (senior program coordinator), Shannon DeGruy, Dorothy Paul, Latasha Barlow, Nicole Bell, Erica Harris (home evaluators); Tulane University Health Sciences Center, Jane El-Dahr (investigator); Tulane University School of Medicine, Maxcie Sikora (physician); Tulane Clinical and Translational Research Center of Tulane and Louisiana State Universities Schools of Medicine, Mary Meyaski-Schluter, Virginia Garrison, Erin Plaia, Annie Stell, Jim Outland, Shanker Japa, Charlotte Marshall (nursing staff); New Orleans Health Department, Kevin Stephens (principal investigator), Mosanda Mvula (investigator), Stacey Denham, Margaret Sanders, Claire Hayes (asthma counselors), Alfreda Porter, Tenaj Hampton, Angela Sarker (community health workers), Mamadou Misbaou Diallo, Shawanda Rogers, David Ali (recruiters), Doryne Sunda-Meya, Ariska Fortenberry (administrative), Florietta M. Stevenson (personnel); Louisiana State University Health Sciences Center School of Nursing, Yvonne Sterling (investigator); Louisiana State University Health Sciences Center, Ken Paris (physician); National Institute of Environmental Health Sciences, Patricia Chulada (health scientist administrator); and National Institute of Child Health and Human Development, William Martin II (principal investigator). Eleanor Thornton (investigator) is employed by Visionary Consulting Partners, LLC, Fairfax, VA. Rich Cohn (investigator) is employed by SRA International, Inc., Durham, NC. Herman Mitchell (principal investigator), Suzanne Kennedy (investigator), John Lim (data manager), Gina Allen (research associate), Jeremy Wildfire, Katy Jaffee, Agustin Calatroni, Becca Zabel, John Schwarz (statisticians), and Theresa Zucchero Scocca (scientist) are employed by Rho, Inc., Chapel Hill, NC.

Supplies were generously donated or discounted: Lincoln Diagnostics, Inc., Decatur, IL, donated the Multi-Test II devices; Greer Laboratories, Inc., Lenoir, NC, donated the allergenic extracts used for skin testing; Ives Business Forms, Inc., New Orleans, LA, provided a discount for environmental supplies; Kaz USA, Inc., Southborough, MA, provided a discount on Honeywell 40200 Platinum HEPA Air Purifier units; Allergy Control Products, Inc., Danbury, CT, provided mattress, box spring, and pillow encasings at cost. Additional funding for environmental and administrative supplies and equipment and for patient transportation vouchers was provided by MCAN.

A variety of committees and working groups were formed for study development and implementation. The Community Advisory Group was critical for recruitment and community outreach. Members included Corey Hebert, pediatrician, Children’s Medical Clinic (chair); Julia Bland, executive director, New Orleans Children’s Museum; Mary Croom-Fontenot, All Congregations Together (ACT); Janice Dupuy, principal, Audubon Montessori Charter School; Stephanie Duplantier, parent; Andrea Duplechain, director of nurses, Algiers Charter Schools Association; Marilyn Hammett, nurse supervisor, Recovery School District; Kathleen Kennedy, dean, Xavier School of Pharmacy; Julie Morial, family physician; Christy Ross, assistant director of Tobacco Free Living; Joe Rossolino, associate superintendent, Archdiocese School System; Diane Russel, superintendent, Jefferson Parish School Board; Beverly Wright, executive director, Deep South Center for Environmental Justice; and Alida Wyler, health services director, Jefferson Parish School Board.

## References

[r1] Blanc PD, Eisner MD, Katz PP, Yen IH, Archea C, Earnest G (2005). Impact of the home indoor environment on adult asthma and rhinitis.. J Occup Environ Med.

[r2] Boudreaux E, Emond S, Clark S, Camargo C. (2003). Race/ethnicity and asthma among children presenting to the emergency department: differences in disease severity and management. Pediatrics.

[r3] Brandt M, Brown C, Burkhart J, Burton N, Cox-Ganser J, Damon S (2006). Mold prevention strategies and possible health effects in the aftermath of hurricanes and major floods.. MMWR Recomm Rep.

[r4] Breslau N, Peterson EL, Kessler RC, Schultz LR (1999). Short screening scale for DSM-IV posttraumatic stress disorder.. Am J Psychiatry.

[r5] Crain E, Walter M, O’Connor GT, Mitchell H, Gruchalla RS, Kattan M (2002). Home and allergic characteristics of children with asthma in seven U.S. urban communities and design of an environmental intervention: the Inner-City Asthma Study.. Environ Health Perspect.

[r6] Douwes J, Pearce N, Heederik D. (2002). Does environmental endotoxin exposure prevent asthma?. Thorax.

[r7] Elliot DB (2009). Understanding Changes in Families and Households Pre- and Post-Katrina. Washington, DC:U.S. Census Bureau.. http://www.census.gov/hhes/families/files/katrina-asa.pdf.

[r8] Erickson S, Iribarren C, Tolstykh I, Blanc P, Eisner M. (2007). Effect of race on asthma management and outcomes in a large, integrated managed care organization.. Arch Intern Med.

[r9] Evans R, Gergen PJ, Mitchell H, Kattan M, Kercsmar C, Crain E (1999). A randomized clinical trial to reduce asthma morbidity among inner-city children: results of the National Cooperative Inner-City Asthma Study.. J Pediatr.

[r10] Gergen PJ, Mitchell H, Lynn H (2002). Understanding the seasonal pattern of childhood asthma: results from the National Cooperative Inner-City Asthma Study (NCICAS).. J Pediatr.

[r11] Grimsley LF, Chulada PC, Kennedy S, White L, Wildfire J, Cohn RD (2012). Indoor environmental exposures for children with asthma enrolled in the HEAL study, post-Katrina New Orleans.. Environ Health Perspect.

[r12] Gruchalla RS, Pongracic J, Plaut M, Evans R, Visness CM, Walter M (2005). Inner City Asthma Study: relationships among sensitivity, allergen exposure, and asthma morbidity.. J Allergy Clin Immunol.

[r13] Henry J. Kaiser Family Foundation (2007). Giving Voice to the People of New Orleans: the Kaiser Post-Katrina Baseline Survey 7631. Washington, DC:Henry J. Kaiser Family Foundation.

[r14] Kates RW, Colten CE, Laska S, Leatherman SP (2006). Reconstruction of New Orleans after Hurricane Katrina: a research perspective.. Proc Natl Acad Sci USA.

[r15] Liu A, Fellowes M, Mabanta M (2006). A One-Year Review of Key Indicators of Recovery in Post-Storm New Orleans. (Special Edition of the Katrina Index).

[r16] Mitchell H, Cohn RD, Wildfire J, Thornton E, Kennedy S, El-Dahr JM (2012). Implementation of evidence-based asthma interventions in post-Katrina New Orleans: the Head-off Environmental Asthma in Louisiana (HEAL) Study.. Environ Health Perspect.

[r17] Morgan WJ, Crain EF, Gruchalla RS, O’Connor GT, Kattan M, Evans R (2004). Results of a home-based environmental intervention among urban children with asthma.. N Engl J Med.

[r18] National Heart, Lung, and Blood Institute (2007). Expert Panel Report 3 (EPR-3): Guidelines for the Diagnosis and Management of Asthma–Full Report 2007. August 28, 2007.. http://www.nhlbi.nih.gov/guidelines/asthma/asthgdln.htm.

[r19] NIEHS (National Institute of Environmental Health Sciences) (2002). National Survey of Lead and Allergens in Housing (NSLAH).. http://www.niehs.nih.gov/research/clinical/join/studies/riskassess/nslah.

[r20] O’Connor G, Walter M, Mitchell H, Kattan M, Morgan WJ, Gruchalla RS (2004). Airborne fungi in the homes of children with asthma in low-income urban communities: The Inner-City Asthma Study.. J Allergy Clin Immunol.

[r21] Rao CY, Riggs MA, Chew GL, Muilenberg ML, Thorne PS, Van Sickle D (2007). Characterization of airborne molds, endotoxins, and glucans in homes in New Orleans after Hurricanes Katrina and Rita.. Appl Environ Microbiol.

[r22] Ratard R, Brown C, Ferdinands J, Callahan D, Dunn K, Scalia M (2006). Health concerns associated with mold in water-damaged homes after Hurricanes Katrina and Rita–New Orleans area, Louisiana, October 2005.. MMWR Morb Mortal Wkly Rep.

[r23] Reynolds S, Black DW, Borin SS, Breuer G, Burmeister LF, Fuortes LJ (2001). Indoor environmental quality in six commercial office buildings in the midwest United States.. Appl Occup Environ Hyg.

[r24] Rudowitz R, Rowland D, Shartzer A. (2006). Health care in New Orleans before and after Hurricane Katrina.. Health Aff (Millwood).

[r25] Rylander R, Lin RH (2000). (1□3)-β-d-glucan–relationship to indoor air-related symptoms, allergy and asthma.. Toxicology.

[r26] Rylander R, Norrhall M, Engdahl U, Tunsater A, Holt PG (1998). Airways inflammation, atopy, and (1□3)-β-d-glucan exposures in two schools.. Am J Respir Crit Care Med.

[r27] Solomon GM, Hjelmroos-Koski M, Rotkin-Ellman M, Hammond SK (2006). Airborne mold and endotoxin concentrations in New Orleans, Louisiana, after flooding, October through November 2005.. Environ Health Perspect.

[r28] Turyk ME, Hernandez E, Wright RJ, Freels S, Slezak J, Contraras A (2008). Stressful life events and asthma in adolescents.. Pediatr Allergy Immunol.

[r29] Vesper S, McKinstry C, Haugland R, Wymer L, Bradham K, Ashley P (2007). Development of an Environmental Relative Moldiness index for US homes.. J Occup Environ Med.

[r30] Vesper SJ, Varma M, Wymer LJ, Dearborn DG, Sobolewski J, Haugland RA (2004). Quantitative polymerase chain reaction analysis of fungi in dust from homes of infants who developed idiopathic pulmonary hemorrhaging.. J Occup Environ Med.

[r31] Wright RJ (2005). Stress and atopic disorders.. J Allergy Clin Immunol.

